# Shared Neuroinflammatory Mechanisms Across Dementia Types: An Integrative Review

**DOI:** 10.3390/ijms27010179

**Published:** 2025-12-23

**Authors:** Subramanian Thangaleela, Asif Ali, Yohanes Tandoro, Chin-Kun Wang

**Affiliations:** 1 Department of Nutrition, Chung Shan Medical University, 110, Sec. 1, Jianguo North Road, Taichung City 40201, Taiwan, China; thangaleela@gm.csmu.edu.tw (S.T.);; 2 Faculty of Agricultural Technology, Widya Mandala Catholic University Surabaya, Surabaya 6026, Indonesia

**Keywords:** dementia, neuroinflammation, Alzheimer’s disease dementia, Lewy body dementia, frontotemporal dementia, vascular dementia

## Abstract

Dementia is a neurodegenerative condition marked by progressive cognitive decline, which affects people worldwide. Studies on dementia typically continue over years of uncertainty. Different types of dementia, like Alzheimer’s disease dementia, Lewy body dementia, frontotemporal dementia, and vascular dementia, exhibit different pathological features, yet their downstream inflammatory pathways involve similar inflammatory mediators. As an initial trigger, microglial cells and astrocytes become activated by protein aggregates, mutations, or any other cause, and release pro-inflammatory cytokines, which can lead to synaptic dysfunction, neuronal degeneration, and impaired cognitive function. Neuroinflammation plays a critical role in the pathogenesis of all forms of dementia. Despite their distinct neuropathological features, inflammatory processes may coincide at a point and lead to neuronal degeneration and cognitive decline. Recent advancements in neuroimaging techniques and biomarker discovery revealed potential therapeutic targets that may mitigate neuroinflammation. The primary objective of this review is to explore the underlying mechanisms linking neuroinflammation to various types of dementia. This review focuses on shared and distinct neuroinflammatory mechanisms to unravel significant therapeutic strategies for dementia.

## 1. Introduction

Dementia is one of the major global threats to public health and is a social care priority. It is characterized by progressive loss or decline in memory. It affects the thinking skills of a person and disturbs the person’s day-to-day life activities [1]. The most common causes of dementia are aging, physical injuries to the brain, stroke, nutritional deficiencies, hypertension, diabetes, obesity, smoking, excessive alcohol consumption, HIV infection, and neurodegenerative conditions like Alzheimer’s disease (AD) [2]. The links between these causative factors and dementia neither work all together nor are linearly associated with the amyloid hypothesis. Aging causes physiological functional decline, which is a profound risk factor for many non-infectious diseases, including AD. Genome-wide association studies (GWAS) revealed the genetic susceptibility of more than 20 risk loci of AD [3]. Head injuries caused by external force also result in the disruption of normal brain functions. Traumatic Brain Injury (TBI) is found to be associated with dementia risk. Moderate and severe TBI are characterized by loss of consciousness and a two-to-four-fold increased risk of dementia compared to individuals without a history of TBI [4]. Our brain health depends on the health of the heart and blood vessels. Conditions like hypertension, obesity, diabetes, and stroke pose cardiovascular risks that are closely related to brain health and increase the risk of dementia. When a stroke hits the brain and the blood vessels are blocked, it markedly induces the loss of neurons and causes dementia [5,6]. Socioeconomic status, related to economic resources, employment, and financial security, comprises the social determinants of health, the non-medical factors that influence the health outcomes, which are associated with various factors such as smoking, alcohol habits, diabetes, hypertension, less physical activity, stress, and depression. These factors work invariably on heart health and eventually cause a threat to cardiovascular health, in turn affecting the brain [4]

The global action plan of dementia 2017–2025, initiated by the World Health Organization (WHO), aims to achieve the betterment of the lives of people with dementia, their caregivers, and their families, in order to reduce the impact of dementia on the community of people related to dementia [7]. The action plan creates and strengthens associations and organizations for people with dementia, their families, and their carers, in order to motivate them and to engage actively, along with healthcare workers and government authorities, in reforming health, policies, and plans relevant to dementia. It supports dementia awareness programs to promote a good quality of life for people with dementia in order to ensure their security and their dignity, as well as that of their families and carers. This action plan helps in aspects such as the quality of life of people with dementia by safeguarding their human rights and protecting them from the social stigma related to dementia in society [7].

Dementia can be broadly defined as a syndrome characterized by a decline in cognitive function that significantly interferes with day-to-day living and functioning. Dementia includes a range of symptoms like memory loss, impairments in reasoning and judgment, and difficulties in decision-making, processing language, and spatial knowledge. The fifth edition of the Diagnostic and Statistical Manual of Mental Disorders classified dementia as a neurocognitive disorder, with various etiological subtypes such as AD, Lewy body dementia (LBD), Parkinson’s disease with dementia (PDD), vascular dementia (VaD), and frontotemporal dementia (FTD). Each type of dementia is characterized by different pathological features, symptoms, disease progressions, treatments, and care needs [8].

For decades, dementia has been defined as a chronic loss of cognitive abilities due to brain injury and disease. Mild cognitive impairment (MCI) is another kind of cognitive weakening defined by a reduced performance or ability to carry out daily activities at home, work, in social settings, as well as to carry out personal care, but it is not consistent with dementia [9]. MCI does not always progress to dementia [10]. Dementia diagnosis requires the complete clinical evaluation of cognitive decline and the examination of mental wellbeing to define impairments in language, attention, visuospatial cognition, memory, and mood status [10,11]. Advanced neuroimaging techniques are now prevailing, which enhance the diagnosis of dementia. Diffusion tensor imaging (DTI), functional MRI (fMRI), single-photon emission computed tomography (SPECT), and PET have been used in dementia diagnosis. The structural connectivity of white matter tracts, changes in brain activity patterns, in blood flow, and in brain metabolism can be studied using these imaging methods [12]. The divergence of dementia challenges the reliable and accurate classifications between the types and complicates clinical diagnosis. Advanced imaging techniques and biomarker analysis may differentiate between the dementia subtypes, ensuring their precise diagnosis, management, and treatment plans.

Dementia risk factors are varied and encompass various genetic and lifestyle influences, cardiovascular conditions, diabetes, obesity, and hypertension. Neuroinflammation is considered the hallmark of neurodegenerative diseases [13] and has been studied extensively. It can be defined as involving multistage physiological responses directed by the reactive immune residents of the CNS, called the glial cells, especially astrocytes, microglial cells, and a cascade of pro-inflammatory factors. These cells and inflammatory factors are triggered by infections, toxins, autoimmunity, protein aggregations of tau or Aβ, and other cell-damaging processes. This protective mechanism initiated by the CNS towards the injury or invasion involves the activation of microglial pattern recognition receptors (PRRs) like toll-like receptors (TLRs) [14,15]. The inflammatory process involves bidirectional communication between the central and the peripheral immune system. Uncontrolled chronic inflammation produces excessive pro-inflammatory cytokines, C-reactive protein, and chemokines, and the dysregulation of neuronal homeostasis, the blood–brain barrier (BBB), oxidative damage, mitochondrial dysregulation, and neurodegeneration [16,17,18]. In AD, LBD, and FTD, neuroinflammation is a central pathological feature. In VaD and other cases of non-medical causes of dementia, such as brain injuries and infections, neuroinflammation occurs, but through different mechanisms. In the latter case, it acts as an amplifier of the neuronal injury rather than an initiating pathology. Therefore, neuroinflammation is common, but its role and driving mechanism differ between the categories. Across most dementias, a detailed discussion of the shared mechanism is essential. This narrative review explores the shared pathophysiological mechanisms of various types of dementias, with particular emphasis on neuroinflammation, and aims to highlight the common molecular pathways and inflammatory signaling that underpin dementia progression.

## 2. Literature Search

A literature search was conducted across multiple databases, including PubMed, Scopus, Web of Science, and Google Scholar. Peer-reviewed articles published within the last two decades were searched. The search keywords included “neuroinflammation”, “dementia”, “Alzheimer’s dementia”, “Lewy body dementia”, “Frontotemporal dementia”, “Vascular dementia”, “pathophysiology of Alzheimer’s disease”, “pathophysiology of Lewy Body dementia”, “pathophysiology of Vascular dementia”, “pathophysiology of fronto temporal dementia”, “Neuroinflammation in Alzheimer’s disease”, “Neuroinflammation in Lewy Body dementia”, “Neuroinflammation in vascular dementia”, “Neuroinflammation in fronto temporal dementia”, “biomarkers in dementia”, “combined molecular mechanisms in dementia”, “shared pathophysiology of dementias”, and “shared mechanisms”. Only articles published in English were included. Relevant in vitro, in vivo, and human studies were reviewed, along with original research articles, systematic reviews, meta-analyses, and narrative reviews. During the screening, special attention was given to studies discussing more than one dementia type or highlighting common pathways, enabling the integration of both disease-specific and shared mechanistic insights in this review.

## 3. Dementia and Its Subtypes

### 3.1. Alzheimer’s Disease Dementia (ADD)

AD is the sixth leading cause of death and the fifth leading cause of morbidity among people over the age of 65 years [10]. Two primary hypotheses of AD are the cholinergic hypothesis and the amyloid plaque hypothesis [19,20,21]. In addition to these hypotheses, various other factors such as age, genetic factors, head injuries, vascular diseases, infections, and environmental factors also contribute to the development of AD [22]. According to recent propositions, there are three clinical phases of AD which include (1) pre-symptomatic AD, which may last for decades until the accumulation of Aβ reaches the threshold level that triggers amyloid cascade in the brain, (2) a pre-dementia phase of AD, which is characterized by amnestic mild cognitive impairment where the pathological degree ranges from mild neuronal pathology to early-stage Braak pathology, this stage may last for several years based on individual resilience, (3) and a clinically defined dementia phase of AD, which involves significant cognitive and functional impairment, along with the accumulation of neuritic plaques and NFT in various brain regions [23].

#### Pathophysiology of ADD

AD is a multifactorial disease with various causes like genetics, aging, environmental factors, cardiovascular diseases, obesity, and diabetes [24]. Pathological manifestations are broadly classified as positive and negative lesions. Positive lesions occur due to the accumulation of NFT, amyloid plaques, dystrophic neurites, neuropil threads, and other depositions. These abnormal structures are called “positive” because they represent something extra that is found during pathology. Negative lesions result from neuronal loss characterized by extensive atrophy resulting from neuronal, neuropil, and synaptic degeneration. As these represent the loss of normal brain structures and reflect a deficit or loss, they are called “negative” [25,26]. Amyloid precursor protein (APP) is a type-1 integral glycoprotein, abundantly expressed in the central nervous system (CNS), which undergoes non-amyloidogenic cleavage by α-secretase to form soluble APPα and an α C-terminal fragment (αCTF). In the amyloidogenic pathway, β- and γ-secretase cleavage generate Aβ40 and Aβ42 peptides, with Aβ42 exhibiting a higher propensity for aggregation and neurotoxicity [23,27]. These aggregates disrupt mitochondrial function, enhance oxidative stress, dysregulate calcium homeostasis, promote tau phosphorylation, impair synaptic plasticity, and ultimately lead to neuronal death [28].

Genetic abnormality or polymorphisms in the gene, such as apolipoprotein E (*APOE*) *ε*4 allele, cause increased amyloidogenic cleavage of APP and reduced Aβ clearance from the brain [29]. Microtubule-associated protein tau (MAPT) becomes hyperphosphorylated, detached from microtubules, and accumulates in the somatodendritic region as paired, helical, and straight filaments during AD development [30]. Tau hyperphosphorylation negatively affects its tubulin binding ability, unsettles the microtubule structure, destabilizes it, impairs the axonal transport, and leads to the formation of NFTs [31]. Genes such as *APP*, Presenilin 1 (*PSEN1*), and Presenilin 2 (*PSEN2*) are associated with autosomal familial AD [32]. Since the discovery of the *APP* gene, approximately 30 mutations have been identified, and more than one-tenth of all early-onset AD (EOAD) cases are accounted for by *APP* mutation [33]. *PSEN1* and *PSEN2* are the components of γ-secretase, which is responsible for Aβ cleavage. A mutation in *PSEN2* is reported to increase the ratio of Aβ42 to Aβ40 in mice and humans [34]. *PSEN1-L166P* mutations result in reduced Aβ production. *PSEN1-G384A* mutations increase the Aβ42. *PSEN2* is less efficient at producing Aβ than *PSEN1* [35]. Mutations in the Triggering Receptor Expressed on Myeloid Cells 2 (*TREM2*) have been found to increase the risk of AD. In addition, there are other genes, including Sortilin-related receptor-1 (*SORL1*), *clusterin* (*CLU*), *complement receptor 1* (*CR1*), and Phosphatidylinositol Binding Clathr in Assembly Protein (PICALM), also linked with late-onset AD (LOAD) [30,36]. The *CLU* gene is responsible for Aβ clearance from the brain, PICALM is involved in communication between nerve cells, *CR1* mutation causes a deficiency in the CR1 protein, which in turn is linked to chronic inflammation [36].

### 3.2. Vascular Dementia (VaD)

VaD is a “disease with a cognitive impairment resulting from cerebrovascular disease and ischemic or hemorrhagic brain injury” [37]. VaD is characterized by cerebrovascular disease, age, sex, atherogenic and other vascular disorders, genetic factors, and inflammation. Other potential factors include obesity, hypertension, hypercholesterolemia, psychological stress, family history of stroke, prolonged alcohol use, diabetes, smoking, and physical inactivity [37,38]. The VaD condition includes different types of vascular damage that affect both small and large blood vessels of the brain, resulting in cerebral hypoperfusion, disruption of the BBB, glymphatic dysfunction, and neuronal damage [39]. Approximately 25–30% of ischemic stroke survivors develop either immediate or delayed vascular cognitive impairment (VCI) or VaD [40]. The term VCI includes a broad spectrum of cognitive disorders ranging from MCI to VaD due to ischemic or hemorrhagic stroke, which entails vascular changes and insidious neurodegeneration [41]. VCI is biologically considered a form of dementia caused by alterations in cerebral blood vessels or cerebrovascular insufficiency, arterial hardening, and failure of blood vessels to deliver blood to the brain [42]. VCI is therefore more widely accepted, with its most severe form recognized as VaD. The combined term, vascular cognitive impairment and dementia (VCID), refers to the cognitive impairment and dementia [43].

#### Pathophysiology of VaD

The most important mechanisms behind VaD pathology are endothelial dysfunction, chronic inflammation, impaired glymphatic clearance, white matter demyelination, and synaptic failure [39]. Vascular lesions and atherosclerosis conditions resulting from hypertension, diabetes, and hyperlipidemia also produce cardiovascular risks and can cause cognitive impairment in VaD patients [44]. Cerebral blood vessels not only play a major role in delivering oxygen and nutrients but also serve as a link between neurons and glial cells. Hence, any damage to these vessels can disrupt vital brain interactions, thereby increasing the risk of vascular and neuronal damage [45]. Cerebral small vessel disease is mainly recognized as a major factor of VaD in the aging population [46]. Recent genome-wide association studies have identified novel genetic risk factors for VaD, including loci such as *SPRY2* (Sprouty RTK signaling antagonist 2), *FOXA2* (Forkhead Box A2), *AJAP1* (Adherens junction-associated protein 1), and *PSMA3* (Proteasome 20S subunit alpha 3), which are linked to hypertension, diabetes, and neuronal maintenance [47]. The well-known heritable cause is Cerebral Autosomal Dominant Arteriopathy with Subcortical infarcts and Leukoencephalopathy (CADASIL). It occurs due to the mutation in the *NOTCH3* gene [48]. The *NOTCH3* gene encodes a transmembrane receptor responsible for vascular smooth muscle cell function [49]. Hereditary cerebral hemorrhage was found to be another hereditary form of VaD in addition to CADASIL [50]. The genetic mutation in the *APP* gene in cerebral vessels can lead to amyloid deposition in these vessels [51]. Mutations in the *COL4A1-A2* gene cause small vessel arteriopathy and intracranial hemorrhage [52]. The clinical manifestations of this disease involve recurrent lacunar strokes along with progressive cognitive impairment, seizures, and psychiatric disturbances [53]. Reduced α-galactosidase A activity leads to the accumulation of glycosphingolipid in various organs. This causes stroke, ischemic attack, renal disease, and cardiomyopathy [54]. Polymorphisms in the Sterol Regulatory Element Binding Transcription Factor (*SREBF-2*) gene influence lipid metabolism, which may be an important factor for VaD [55]. A case–control study carried out in Koreans with VaD and healthy subjects showed that single-nucleotide variants of the vascular endothelial growth factor (*VEGF*) gene are also associated with VaD [55]. Amyloid pathology and VaD pathology are linked by the *APOE ε4* allele. Mutation in *APOE ε4* impairs glymphatic function [56] and increases the risk of post-stroke dementia (PSD), exacerbates BBB disruption, and produces cerebral amyloid angiopathy (CAA) [57,58]. Polymorphisms in methylenetetrahydrofolate reductase (*MTHFR*) genes result in hyperhomocysteinemia, a known risk factor for vascular damage, endothelial dysfunction, and oxidative stress found in VaD and small vessel disease patients [59]. Polymorphic variants of IL-6 and TNF-α genes were also found to be associated with VaD risks [60].

Amyloid angiopathy inflicts damage to cerebral tissues, subcortical white matter, leading to damage of oligodendrocytes, demyelination, axonal injury, disruption of the neural network, and cognitive disturbances [61,62]. VaD also encompasses a wide range of cerebrovascular pathologies, which can likely be defined as a spectrum of cerebrovascular pathologies, including the PSD, subcortical ischemic vascular dementia (SIVD), hereditary forms, and CADASIL. These conditions produce excitotoxicity, peri-infarct inflammation, glial scars, circuit disruption [63], chronic hypertension, white matter hyperintensities, cerebral microbleed, enlarged perivascular spaces, reduced vascular compliance, and cerebral blood flow [64,65,66]. The vascular risk factors trigger the endothelial cells to upregulate vascular cell adhesion molecule-1 (VCAM-1) and intracellular adhesion molecule-1 (ICAM-1), which facilitate leukocyte infiltration into the brain parenchyma [67]. Also, the tight junctions between endothelial cells are altered by the matrix metalloproteinase-9 (MMP-9), released by the activated endothelial cells and astrocytes [68].

### 3.3. Lewy Body Dementia (LBD)

Lewy body dementia (LBD) includes Parkinson’s disease dementia (PDD) and dementia with Lewy bodies (DLB), both of which are characterized by accumulation by the accumulation of Lewy bodies (LB) [69,70]. In PDD, Parkinsonian features occur first, and dementia occurs later. In DLB, dementia occurs first, followed by Parkinsonism occurring later. LBD was considered an underdiagnosed disease due to overlapping clinical symptoms with PD and AD [71]. LB are abnormal inclusions made of aggregates of α-synuclein within neurons found in PD, LBD, and DLB. The synucleinopathies refer to PD, PDD, LBD, and multiple system atrophy (MSA), which are also characterized by the accumulation of α-synuclein proteins [72]. LBD is characterized by various cognitive, neuropsychiatric, sleep, motor, and autonomic symptoms [73]. Clinical studies state that among dementia patients, approximately 4–8% of cases are attributed to LBD, and up to 80% of PD patients are found to have dementia [70,71]. Both PDD and LBD share similar clinical features, making it difficult to distinguish between the two conditions without further assessment [74]. The “1-year rule” is commonly applied to differentiate between diagnoses: if dementia onset occurs within 12 months of Parkinsonism, it is classified as DLB, whereas if Parkinsonism symptoms appear within 12 months after the onset of dementia, it is classified as PDD [75]. Additionally, mixed pathologies can occur, with AD patients exhibiting LB accumulation alongside amyloid plaques and neurofibrillary tangles (NFTs) [76]. The amygdala, periamygdaloid regions, and entorhinal cortex are frequent sites of LB deposition, although LB pathology may also extend to the neocortical and brainstem regions in AD cases [77].

#### Pathophysiology of LBD

The pathophysiology of LBD is characterized by aggregation of misfolded α-synuclein referred to as Lewy bodies (LBs), which cause neuronal degeneration and neuronal death [78]. Under physiological conditions, α-synuclein is involved in regulating the synaptic vesicle cycle at presynaptic terminals [79]. However, when α-synuclein undergoes pathological modifications such as phosphorylation, truncation, or nitration, it tends to aggregate into fibrils, leading to dysfunction of dopaminergic and cholinergic neurons—a central feature of LBD pathology [80]. In PD, LB accumulation is prominent in the substantia nigra, but in LBD, significant deposition is also observed in cortical regions [81]. Several studies suggest that α-synuclein possesses prion-like properties, enabling its cell-to-cell transmission, thereby promoting aggregation in recipient neurons and accelerating neurodegeneration [82]. Genetic factors include mutations in the alpha-synuclein (*SCNA*) gene, which is a major component of LB [83]. Mutations in the *GBA* gene that encodes the enzyme glucocerebrosidase are important contributors to LBD. Individuals with *GBA* gene mutation often show severe cognitive decline and a higher susceptibility to develop dementia [84,85]. Individuals with LBD who carry the *APOE* ε*4* allele may exhibit more cognitive impairment and a higher burden of AD pathology as well [86]. Other mutations in genes *PSEN1* and *PSEN2*, which are primarily associated with AD, have also been found in some LBD cases. These mutations produce overlapping clinical features in patients with both AD and LBD [87,88]. *CHMP2B* and *SQSTM1* are additional gene variants that are rarely observed in some cases [88]. Genetic risks, such as mutations in *SNCA* and leucine-rich repeat kinase 2 (*LRRK2*) genes, have also been identified in LBD [89,90]. α-synuclein is not only involved in LBD and PDD but also acts as an important etiological factor for other α-synucleinopathies, including MSA [91]. Neurotransmitter imbalances also play a significant role in LBD pathophysiology. Degeneration of cholinergic neurons in the basal forebrain results in reduced acetylcholine levels, which can cause cognitive decline and visual hallucinations [92]. Acetylcholine deficiency is likely more pronounced in LBD and prominent feature of LBD, and more severe than in PD. Postmortem studies have revealed that brain regions, including the substantia nigra, dorsal raphe, locus coeruleus, and the dorsal motor nucleus of the vagus nerve with reduced dopamine levels. In addition to the α-synuclein protein, ubiquitin and neurofilament proteins are also associated with LBD pathology.

### 3.4. Frontotemporal Dementia (FTD)

Frontotemporal dementia (FTD) shows a spectrum of clinical manifestations characterized by progressive behavioral and language disturbances. FTD mostly affects middle-aged individuals between 45 and 65 years [93]. Intracellular deposition of TAR DNA binding protein (TDP-43) aggregates in the frontal and temporal lobes of the brain, resulting in frontotemporal lobar degeneration, microvacuole formation, and astrocytosis [93,94]. FTD affects the brain areas responsible for language learning, behavior, personality, and motivation. Changes in behavior and difficulty in language are the initial clinical features, followed by cognitive loss [95]. FTD shows different types of neuropathology, mainly degeneration of the frontal and temporal lobes. Also, abnormal inclusions of tau and TDP-43 protein have been identified in many cases [96]. FTD patients are closely linked to amyotrophic lateral sclerosis (ALS) either clinically or through genetic similarities [97]. A wide range of clinically and pathologically related disorders, including ALS, bvFTD, non-fluent agrammatic variant primary progressive aphasia (nfvPPA), semantic variant PPA (svPPA), and logopenic variant PPA (lvPPA), are grouped under FTD, but each type has clear diagnostic criteria [98,99]. In FTD, the most prevalent clinical syndrome is bvFTD, characterized by behavioral changes and atrophy of the frontal and anterior temporal lobes. Clinical diagnostic criteria involve social disinhibition, emotional blunting, such as a lack of sympathy and empathy, dietary changes including a preference for sweetened foods, and repetitive, obsessive, stereotyped behaviors [100]. Some patients also show psychotic symptoms of delusions and hallucinations [101,102,103]. svFTD is primarily a disorder of conceptual knowledge characterized by confused or impaired understanding of the meaning of words, objects, faces, and other sensory stimuli, and is associated with language-related problems [104,105]. svFTD shows bilateral, asymmetrical atrophy of the temporal lobes [104]. The nfvPPA is another clinical syndrome characterized by impaired language and deficient grammar usage associated with asymmetrical atrophy of the left hemisphere. Speech disorders are not present in all patients, but some patients show distorted utterances such as speech apraxia [106,107]. Pathologically, histological evidence reveals abnormal neuronal and glial cells, along with aggregated tau proteins, and cytoplasmic tau inclusions in neuronal cells [108].

#### Pathophysiology of FTD

Neuropathological signs of FTD include frontotemporal lobar degeneration with tau and TDP-43 accumulation. FTD is associated with movement disorders and abnormalities, including akinetic rigidity, corticobasal syndrome, progressive supranuclear palsy, and/or motor neuron disease FTD [109,110]. Familial FTD is present in approximately 25–50% with autosomal-dominant inheritance showing mutations in *MAPT*, *GRN*, chromosome 9 open reading frame 72 (*C9orf72*), valosin-containing protein (*VCP*), and charged multivesicular body protein 2B (*CHMP2B*) [111]. The pathophysiology of FTD involves various genetic mutations, protein misfolding, and neuroinflammatory processes [112]. Familial FTD cases occur due to mutations in the microtubule-associated protein tau (*MAPT*) [113], which lead to an imbalance in tau isoforms and misregulation of tau protein splicing, resulting in overproduction of tau, which accumulates to form NFTs [114]. In addition to tau pathology, accumulation of TDP-43 protein in the cytoplasm of neurons also contributes to neurodegeneration [115]. The other common gene mutations in FTD include chromosome 9 open reading frame 72 (*C9orf72*) and *progranulin* (*GRN*) [116]. Patients with *C9orf72* gene mutations often exhibit psychiatric symptoms, including delusions, hallucinations, and obsessive-compulsive or psychotic features due to mild and symmetric atrophy of the dorsal frontal, temporal, parietal, cerebellar, and dorsomedial thalamic regions [116]. Pathology due to mutation in the *C9orf72* gene is characterized by accumulation of dipeptide repeat proteins and TDP-43 inclusions in the brain [117,118]. Mutations in the *GRN* gene lead to haploinsufficiency, where a single copy of the *GRN* gene is insufficient to maintain normal levels of progranulin protein [119]. Loss of progranulin protein causes neuroinflammation and neurodegeneration, particularly in the frontal and temporal lobes, leading to difficulties in behavior and language [120]. Clinical manifestations of different types of FTD widely vary. Patients with bvFTD exhibit changes in personality, social conduct, and difficulty in day-to-day activities. svFTD patients are characterized by language deficits [121,122,123]. Neuroimaging studies have shown that individuals with the *C9orf72* mutation exhibit symmetry patterns of atrophy in frontal, temporal, and parietal lobes, in contrast to asymmetric atrophy observed in *GRN-mutated* individuals [117,122]. Patterns of brain atrophy observed in the *GRN* mutation are larger than those in the *C9orf72* mutation. *C9orf72* mutation carriers have smaller overall brain volumes [123]. The difference in brain atrophy reflects distinct pathophysiological mechanisms driven by these mutations [124]. Both *C9orf72* and *GRN* mutations play critical roles in FTD pathogenesis. FTD causative mutations are found in several genes involved in neuroinflammation. Studies revealing the neuroinflammatory and immune-related mechanisms in FTD represent a promising avenue in identifying diagnostic markers and treatment targets for FTD. Different types of gene mutations involved in neurodegeneration and dementia are summarized in Table 1.

The listed neurodegenerative diseases involve the formation of protein aggregates, including the Aβ and NFTs. These pathologies are common, but differ in their distribution, composition, and associated cellular changes. Aβ, NFTs, TDP-43, LB, and cerebrovascular lesions are the features of AD, FTD, LBD, and VaD. Protein inclusions are a common hallmark of neurodegenerative disorders; the type of aggregated proteins, their localization, and the specificity of the brain regions help to distinguish the pathology between the diseases. Despite the convergence in neuroinflammation, rather significant differences also existed between these dementias in terms of neuronal loss, impaired neuronal energy metabolism, disturbed mitochondrial function, and involvement of brain regions.

## 4. Neuroinflammation in Dementia

Neuroinflammation is a key factor in contributing to the cognitive decline in dementias. Upon activation, microglia release pro-inflammatory cytokines such as IL-1, IL-6, and TNF-α, along with chemokines including CCR3, CCR5, and CCL12 [89,147]. Chronic inflammation can cause damage to the BBB and make it leaky as the brain ages, and the inflammatory cells can cross the BBB and enter the brain. This breaching further induces the microglia to liberate more inflammatory responses that cause hyperphosphorylation of tau proteins and NFT formation [148,149]. The formation of NFT releases exosomes that activate pro-inflammatory responses, cytokine release, and NLRP3 complexes. Activated NLRP3 complex produces IL-1β, which binds to IL-1β receptor and initiates neuroinflammation cascades and dementia [122]. The inflammatory mediators can cause alterations in IL-1β, TNF-α, and mitogen-activated protein kinases (MAPKs), which result in changes in the neuronal proteins CREB and BDNF. Thus, alteration in these neurochemicals manifests cognitive dysfunction and dementias [150].

### 4.1. Neuroinflammation in Alzheimer’s Disease

Among immune cells of the CNS, microglia play an important role in orchestrating neuroinflammation. In addition to microglial cells, astrocytes, oligodendrocytes, lymphocytes, and peripheral myeloid cells contribute to neuroinflammation in AD. Generally, microglial cells exist in a homeostatic state and switch to a reactive state when activated or responding to inflammatory stimuli. Microglia, resident macrophages, become activated early in AD, increase in number, and are closely associated with Aβ plaques, NFTs, and complement factors. The activated microglia generate immune mediators such as cytokines, chemokines, inflammasomes, and ROS [13]. Aβ fragments induce an inflammatory response in the brain and express IL-1β and TNF-α receptors. Microglial activation by toll-like receptors (TLRs) and NOD-like receptors (NLRs) induces production of TNF-α, IL-1, and IL-6 [151,152]. The integrity of the BBB is affected by TNF-α, IL-1, and IL-1β by modulating the tight junctions in endothelial cells and thereby allowing leucocytes into the brain [153]. Activated microglia migrated to Aβ plaques and phagocytosed them. Prolonged activation of microglia causes microgliosis, which results in sustained pro-inflammatory cytokine release [154]. The disturbances in the meningeal lymphatic system and glymphatic system reduce the clearance of Aβ, immune mediators, and accelerate neuroinflammation.

Quantitative in vivo measurement of glial activation in normal and AD patients using positron emission tomography (PET) showed that microglial activation is an early event in the pathogenesis of the disease [155]. In AD neuroinflammation, IL-1 acts as a driving force in the diffusion of neuritic plaques and their spread across the cerebral cortex [156]. IL-1β overexpression in the AD mouse model develops amyloid deposition as well as increased p38 MAPK and glycogen synthase kinase-3β, which contribute to tau protein phosphorylation [157,158]. Research shows that TNF-α converting enzyme in the CSF of MCI and AD subjects is significantly higher than in healthy subjects. Significant correlations between CSF Aβ/tau ratios and CSF TNF-α converting enzyme strongly confirm the involvement of TNF-α in AD-related neuroinflammation [159]. TNF-α is an important pro-inflammatory cytokine in AD, and increased TNF-α production has been observed in the brain and plasma of AD patients [160]. TNF-α receptors 1 and 2 were detected at high levels in the CSF of MCI patients who later progressed to AD over six years [161]. TNF-α is produced through activation of the NF-κB transcription factor. TNF-α increases the production of enzyme β-secretase and γ-secretase, thereby enhancing Aβ production [162]. IL-1β regulates the APP synthesis and amyloidogenic pathway of APP by increasing the γ-secretase activity [163]. IL-6 has a dual function, acting as both pro-inflammatory and anti-inflammatory depending on its concentration and context of release [164]. IL-10 and transforming growth factor-β (TGF-β) are involved in anti-inflammatory response and immunosuppression. These proteins are found in healthy brain tissues but remain upregulated in AD patients [165]. TGF-β is a neuroprotective protein that inhibits Aβ production and deposition, suppresses neuroinflammation, and inhibits the tau-phosphorylating enzyme GSK-3β [166]. Reactive astrocytosis upregulates Glial fibrillary acidic protein (GFAP), vimentin, and increases the expression of immune genes [167]. Astrocytes secrete pro-inflammatory cytokines, ROS, including hydrogen peroxide, and stimulate neuronal dysfunction. Crosstalk between microglia and astrocytes perpetuates synaptic toxicity, oxidative injury, and accelerates neuronal degeneration [13].

### 4.2. Neuroinflammation in Vascular Dementia

Neuroinflammation arising from acute or chronic cerebrovascular injury is a primary reason for VaD. Metabolic stress due to hypoperfusion overproduces ROS and reactive nitrogen species (RNS) that overwhelm the brain’s antioxidant defense systems and produce superoxide dismutase, catalase, glutathione peroxidase, which cause cellular damage and inflammation. The oxidative stress markers are elevated in the CSF and brain of VaD patients [168,169]. The immunological responses disturb endothelial functions at the cerebrovascular level, rupture the BBB, facilitating the entry of inflammatory mediators into the brain parenchyma. Thus, the compromised BBB amplifies the inflammation, which causes damage to white and gray matter [170]. Microglia and astrocytes are activated by ischemia and hypoxia [171]. Neuroinflammation harms the brain cells, disrupts the tight junctions between vascular endothelial cells, and weakens the cerebral blood flow in the brain [170]. Activated microglia release inflammatory markers and cytokines such as IL-1β, IL-6, and TNF-α. The prolonged activated microglia cause oxidative damage, mitochondrial dysfunction, axonal and neuronal damage, resulting in further cognitive decline [172]. Increased production of inflammatory cytokines has been observed in the hippocampus and white matter during the chronic stage [173]. In chronic cerebral hypoperfusion (CCH) animal models, neuroinflammation produced damage to the white matter and showed expression of matrix metalloproteinase 2 (MMP2) [174,175]. Increased MMP2 causes angiogenesis and induces BBB damage. MMP2 inhibition reduced BBB disruption, microglial activation, and white matter lesions [176]. VaD coexists with AD. AD patients showed vascular injuries, including cerebral infarction, vascular dysfunction, atherosclerosis, and cerebral myeloid vascular conditions [170]. Peripheral monocytes and neutrophils infiltrate into the brain through compromised BBB and release IL-17, myeloperoxidase, and elastase, causing vascular leakage and neuronal loss [177]. Excessive inflammation is a major contributor to the onset and development of VCID. Ischemic and hypoxic environments induce neuroinflammation, oxidative stress, BBB damage, neurovascular injury, loss of neuronal function, decrease in cerebral blood flow, and neuronal cell death [178]. Increased production of inflammatory mediators such as IL-1β, IL-6, and TNF-α in the CCH model aggravates ischemic injury [179]. Failure of glymphatic clearance speeds up amyloid deposition, causes accumulation of wastes, inflammatory mediators, and DAMPs, and causes neuroinflammation [180].

### 4.3. Neuroinflammation in Lewy Body Dementia

Neuroinflammation in LBD is primarily driven by the chronic activation of microglia by α-synuclein aggregates and sustained release of pro-inflammatory cytokines such as TNF-α, IL-1β, and IL-6. The immune response is activated by the pattern recognition receptors (PRRs) in the CNS by means of pathogen-associated molecular patterns (PAMPs) or danger-associated molecular patterns (DAMPs). TLR1, TLR2, TLR4, and myeloid differentiation factor 88 (MyD88) play key roles in α-synuclein recognition and subsequent inflammatory signaling cascades [181,182]. It was found that α-synuclein induces pro-inflammatory signals by activating TLR1/2 at the cell membrane, resulting in nuclear translocation of NF-κB, thereby enhancing the production of TNF-α and IL-1β in a MyD88-dependent manner [181]. Involvement of TLR2 in inflammatory response was confirmed by the complete elimination of cytokine and chemokine release in TLR2 knockout mice, but in TLR4 knockout mice, the pro-inflammatory cytokine release remained unaffected. This shows that the involvement of TLR2 in neuroinflammation is related to LBD [183]. Blocking TLR1/2 heterodimer with CU-CPT22 small molecule inhibitor reduces nuclear translocation of NF-κB and secretion of TNF-α in primary mouse microglial cell cultures [181]. Major histocompatibility complex (MHC) class II proteins play a vital role in the antigen presentation and activation of CD4+ T cells in response to α-synuclein. Microglial cells expressing MHC class II interact with the antigen-presenting cells and produce T-cell proliferation and release pro-inflammatory cytokines, which further amplify neuroinflammation and neuronal toxicity [184]. In a mouse model study, full-length human α-synuclein induces MHC class II expression in microglia; meanwhile, an MHC class II knockout mouse model is protected against α-synuclein-induced microglial activation, antigen presentation, IgG deposition, and degeneration of dopaminergic neurons [184].

### 4.4. Neuroinflammation in Frontotemporal Dementia

With the help of imaging studies, it was shown that neuroinflammation is an integral part of FTD pathology [185,186]. PET studies have confirmed neuroinflammation and protein aggregation in bvFTD patients [168,186]. Evidence from genetic studies, epidemiology studies, post-mortem analyses, and animal studies strongly establishes the involvement of innate immune responses in FTD [187,188,189,190]. Immune activation in FTD is due to the aggregation of Aβ, abnormal conformations of tau or TDP43 proteins, or signals released by damaged neurons [191,192]. Several complement proteins were found to be elevated in the CSF and plasma of the 9759genetic FTD patients during symptomatic stages. Clinical and neuroimaging data obtained from genetic FTD cohorts showed that a broad range of complement proteins, like C1q and C3b in CSF, C2 and C3 in plasma, are activated in conjunction with neuronal loss [193]. SvPPA patients showed elevated levels of TNF-α in the plasma and CSF of FTD patients. Elevated TNF-α levels in sporadic and familial FTD patients indicated the involvement of immune responses in both genetic and non-genetic forms of FTD [194]. Plasma and CSF complement proteins indicate that neuroinflammatory response is not merely a consecutive reaction of neurodegeneration but a main contributor to the disease [193]. Phagocytic microglia have been identified clustered around the blood vessels in the hippocampus of a patient affected by FTD associated with *MAPT* mutations [195]. Another study identified more amoeboid microglia in the frontal lobe gray matter, and dystrophic microglia present in the white matter [188]. In the therapeutic aspect, neuroinflammation acts as a promising avenue for intervention in FTD. Strategies such as clearing neurotoxic proteins and inhibiting pro-inflammatory signals might reduce disease progression [196].

## 5. Shared Pathophysiological Mechanism of Dementias

**Figure 1:**
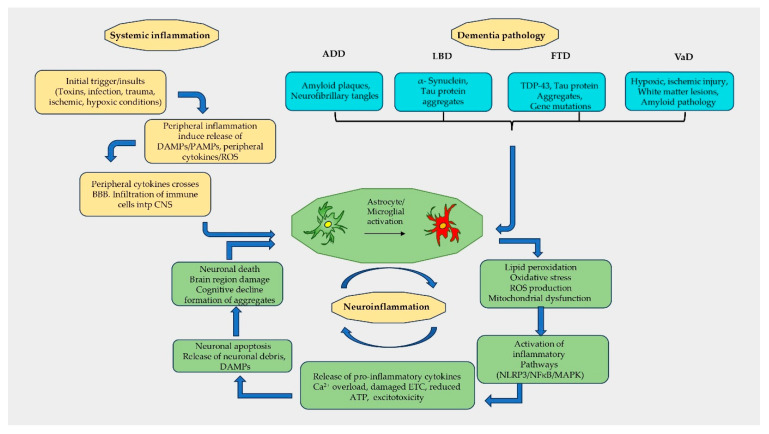
Shared pathophysiology and neuroinflammatory process of different types of dementias. Initial systemic insults such as toxins, infections, trauma, ischemia, or hypoxia initiate peripheral inflammation, leading to the release of damage-associated molecular patterns (DAMPs), pathogen-associated molecular patterns (PAMPs), cytokines, and reactive oxygen species (ROS). These mediators may disrupt the blood–brain barrier (BBB), enabling peripheral cytokines and immune cells to infiltrate the central nervous system (CNS). Amyloid plaques, neurofibrillary tangles, tau and α-synuclein aggregates, gene mutations, and ischemic lesions activate astrocytes and microglia. This activation drives chronic neuroinflammation, further exacerbating neurodegenerative processes and systemic immune responses, thereby creating a self-perpetuating pathological cycle that underlies the progression of dementia.

The pathophysiology of AD, LBD, FTD, and VaD is increasingly recognized as a convergent mechanism mediated through shared neuroinflammatory pathways (Figure 1). Dementia commonly involves the accumulation of specific misfolded protein aggregates, leading to varying degrees of neurodegeneration. Inflammatory signaling pathways, including TLR, TNF, and NF-κB pathways, are shared features across dementias [197]. Neuroimaging studies have demonstrated increased microglial activation and similar neuroinflammatory profiles in AD, LBD, and FTD [198,199], supporting the hypothesis that inflammation is not merely a secondary response but also a critical cross-disease mechanism. Reactive microgliosis, oxidative damage, and mitochondrial dysfunction are implicated in the pathogenesis of all types of dementias [199]. Postmortem analyses of neurodegenerative types of dementia have revealed activated microglia in brain regions specific to the type of dementia [200]. In all dementias, an initial pathologic insult triggers an inflammatory response and neuronal dysfunction. Subsequently, this systemic inflammation promotes further cytokine production, which can cross the damaged BBB and affect neuronal cells [199]. The perturbations in mitochondria induce chronic inflammation through NLRP3 inflammasome-dependent inflammatory pathways [201] and significantly lead to neurodegeneration [202].

In AD, amyloid oligomers, fibrils, and APP activate microglia [203,204], whereas in LBD and PDD, α-synuclein fibrils serve as the primary triggers of microglial activation [205]. In mouse models of FTD, aggregated tau proteins elicit similar inflammatory responses [206]. Progranulin deficiency leads to proteostasis impairment, lysosomal dysfunction, and cytoplasmic TDP-43 aggregation, which induces robust neuroinflammatory responses and shows mechanistic convergence with the pathogenic cascades observed in AD and LBD [207]. Another important biomarker, BDNF, is reduced in the serum of patients with AD, LDB, FTD, and VaD. BDNF is a neurotrophic factor that facilitates neuronal survival and plasticity. Both BDNF mRNA and protein were found to be reduced in multiple brain regions of AD and PD patients’ brains. Thus, the involvement of BDNF in dementias suggests that the BDNF pathway may be linked to neurodegeneration [208]. Neuroinflammation results in the loss of neurons from the hippocampal CA1 region in AD [12] and loss of dopaminergic neurons from the substantia nigra region of the brain in PD models [209]. Activation of pathways such as MAPK and PPAR-γ further contributes to progressive neuronal loss in both AD and PD [199]. PET imaging demonstrates that microglia overexpressing the translocator protein (TSPO) accumulate around senile plaques in the cortex and limbic regions of AD brains [210]. Using the TSPO radioligand [11C] (R) PK11195 (1-(2-chlorophenyl)-N-methyl-N-(1-methylpropyl)-3-isoquinoline carboxamide), activated microglial cells in cortical areas of AD and MCI patients have also been detected [90,91]. Similarly, [11C] (R) PK11195 PET studies revealed higher microglial activation and TSPO binding in the substantia nigra and putamen regions of PD patients with dementia [9,55,92] and in the frontotemporal cortex and basal ganglia of FTD patients [147]. Collectively, these studies indicate that neuroinflammation in ADD, LBD, FTD, and VaD involves shared mechanisms including microglial activation, inflammatory pathway activation, and neuronal damage [199].

Lysosomal dysfunction occurs in AD and PD due to mutations in the genes *APOE*, *PSEN1*, *APP*, *GBA*, *LRRK2*, and *ATP13A2*, respectively [211]. Lysosomal accumulation in LBs is a prominent characteristic of PD neuropathology [212]. In case of FTD, mutations in the genes *MAPT*, *GRN*, *C9orf72*, and transmembrane protein 106B (*TMEM106B*) produce mutated proteins that localize in the lysosome and cause lysosomal dysfunction by altering lysosomal fusion, cargo trafficking, and lysosomal acidification, thereby modulating autophagy [211]. A mutation in the *GRN* gene, which encodes progranulin, causes FTD-GRN through progranulin haploinsufficiency and is additionally linked to AD and PD. Progranulin deficiency is therefore considered a common feature of FTD, AD, PD, ALS, and normal brain aging [213]. Alterations in microglial membrane innate immune receptor TREM2 are also linked to multiple neurodegenerative disorders. The *TREM2* heterozygous *R47H* variant increases risk for developing AD and FTD by impairing TREM2-mediated phagocytosis and responses to neuronal injury, potentially reducing Aβ clearance.

*TREM2* variants have been associated with AD, PD, and FTD. CSF TREM2 levels decreased with AD and FTD [214], whereas they increased in PD patients [215]. Mitochondrial abnormalities in the brain are considered early pathological changes in AD, PD, and FTD [216]. Clinical symptoms like language impairment, cognitive and behavioral changes in FTD also overlap with several other conditions, including frontotemporal lobar degeneration, progressive supranuclear palsy, corticobasal degeneration, and AD [217,218,219]. The person with clinical AD shows early asymmetric frontal and temporal hypometabolism that is comparatively more advanced in a person with bvFTD [220].

Cerebral inflammation is an important etiological factor for the AD progression but is not yet well understood in the case of LBD. To evaluate the neuroinflammatory profile of the cerebral cortex of LBD patients, Amin and team [221] performed a large cohort of human post-mortem study including 30 confirmed LBD cases and 29 matched controls. The team immunolabelled and quantified proteins like α-synuclein, Aβ, phosphorylated tau, microglial phenotype markers Iba-1, HLA-DR, CD68, CD64, CD32a, CD32b, CD16, anti-inflammatory markers IL4R, CH13L1. The results of the analysis showed that the markers exhibited no significant changes in LBD cases compared to controls; furthermore, LBD cases were characterized by T-lymphocyte recruitment in the absence of microglial activation [222]. In cases with co-pathology of AD and LBD, morphologically reactive amoeboid microglia were observed in common. While resting microglia with small cell bodies and thin processes were seen in LBD cases, highly dense, swollen astrocytes were seen in AD cases. The LBD and AD co-pathology cases with higher CD68 loads were found in the amygdala and parahippocampal gyrus. The study concluded that LBD microglial activation is associated with AD co-pathology [223]. The common and shared pathophysiological features of different kinds of dementia are charted in Table 2.

Neuroinflammation serves as a shared pathogenic backbone across AD, LBD, VaD, and FTD. But the initial triggers, regional patterns, and cellular profiles differ. These variations not only shape the clinical phenotype but also influence therapeutic responsiveness and develop the need for dementia-specific as well as inflammation-targeted interventions.

## 6. Biomarkers of Neuroinflammation

### 6.1. Neuroimaging

Diagnosing cognitive impairments and dementia is a major challenge in clinical practice. Brain pathology can be assessed by molecular imaging techniques such as PET, which aid in the temporal and spatial analysis of the living human brain. Microglial reactivity can be traced by using radiopharmaceuticals that target TSPO, found in the outer mitochondrial membrane of microglia [224]. TSPO expressions can also be traced in other cells, like astrocytes and endothelial cells [225]. More recent techniques help trace more microglial target receptors such as P2X7R, P2Y12R, and CX_3_C-chemokine receptor 1 (CX_3_CR1) [226,227]. Supporting the evidence, a longitudinal study of 2.7 years in patients with AD showed a greater increase in TSPO binding in several brain regions compared to controls. This indicates enhanced reactivity of microglial cells and is correlated with cognitive decline [228]. Identifying reliable blood-based biomarkers can reflect pathophysiological mechanisms and offer various advantages compared to brain imaging markers and CSF markers [229]. Neuroimaging studies revealed pathology markers of VaD include white matter hyperintensities and white matter microstructural integrity [65].

### 6.2. Fluid Markers

Fluid biomarkers are reported in blood, and CSF plays an important role in pathogenesis. These include pro-inflammatory, anti-inflammatory cytokines, chemokines, and secondary messengers that coordinate immune responses [39]. Certain proteins, such as TREM2 and YKL-40, act as markers of microglial and astroglial activation; GFAP is the marker of astrocyte reactivity [230,231]. Novel immunoassays allow detection of inflammasome components such as NLRP3, apoptosis-associated proteins in AD [232,233]. MCI patients are at an increased risk of progressing this condition to dementia. But the challenge lies in predicting which patient will eventually develop dementia [234]. Hippocampal volume reduction is observed in MCI patients who later develop AD or VaD [235,236]. MCI patients are differentiated from MCI with underlying AD with the help of CSF biomarkers such as Aβ_42_, phosphorylated tau (P-tau181), and total tau (T-tau) [237]. Other combinations of markers, such as hippocampal atrophy with CSF biomarkers, as well as their combination with neuropsychological test performances, are being studied. The structural imaging on magnetic resonance is important for the clinical assessment of patients suspected of ADD. Atrophy in medial temporal structures is also a valid diagnostic marker [235].

Other endogenous signals are PAMPs and DAMPs, which arise from cellular stress and metabolic imbalance. PRRs are mostly present in the microglia and astrocyte cells in the CNS and support innate immunity. Activated PRRs increase the expression of pro-inflammatory genes and activate the inflammatory caspase 1 [238]. Nucleotide-binding and oligomerization domain-like receptors recognize the PAMPs and DAMPs and trigger the formation of the inflammasome [239]. Continuous activation of PAMPs and DAMPs results in neuroinflammatory reactions and neurodegeneration. The produced cytokines form a feedback loop, activating astrocytes and microglia [240]. Aβ oligomeric fibrils act as DAMPs and initiate inflammasome activation. NLRP3 activation was detected in brain samples of patients suffering from MCI due to AD [241]. In AD, although microglia remove Aβ plaques by phagocytosis, accumulated Aβ triggers the production of inflammatory mediators, which cause neuronal damage and activate intracellular NLRP3 inflammasome [240]. NLRP3 activation is crucial for the pathology and progression of AD. Preventing NLRP3 and inflammatory cytokines activation may help reduce the Aβ deposition and tau protein phosphorylation and ameliorate behavior abnormalities in AD patients [242]. In PD, α-synuclein aggregation activates the NLRP3 inflammasome. Histological studies conducted in PD patients revealed NLRP3 expression in mesencephalic neurons [243]. It was clearly found that the NLRP3 inflammasome is involved in the pathophysiology of both AD and PD. Inhibiting the NLRP3 inflammasome pathway might potentially become a significant therapeutic target in neurodegeneration studies. Serum progranulin levels show high specificity for distinguishing FTD patients associated with *GRN* mutations from other FTD subtypes [244]. TREM2 levels in CSF are a marker of microglial activation in FTD patients. The *GRN* mutation carriers were found to have higher CSF concentrations of TREM2 than *MAPT* or *C9orf72* mutation carriers [245]. Key fluid biomarkers include CSF markers such as NfL [246], the CSF/serum albumin ratio [247], GFAP, YKL-40, TREM2 [248], the Aβ42/40 ratio [249], and phosphorylated tau (p-tau 181, 217) [249]. Plasma biomarkers that parallel these findings include NfL [250], GFAP [251], plasma p-tau [249], and the plasma Aβ42/40 ratio [249]. To clarify the clinical applicability of different biomarkers, we categorized (Table 3) the markers according to ex vivo (on blood or CSF) in living individuals and post-mortem analysis (from brain tissue examination).

There exist significant correlations between inflammatory markers, clinical symptoms, disease progression, and treatment response. Elevated levels of markers are associated with cognitive impairment, behavioral changes, and functional decline. Inflammatory markers can reflect treatment response. Cytokine levels are reduced with anti-inflammatory therapies.

## 7. Future Directions and Therapeutic Relevance in Dementia Research

Dementias, including AD, VaD, LBD, and FTD, share major pathological processes like chronic inflammation, abnormal protein aggregation such as Aβ, tau, α-synuclein, mitochondrial dysfunction, impaired proteostasis, and vascular damage. The emerging therapeutic directions are focused on both individual and shared mechanisms to enable the possibility of a multi-dementia approach.

### 7.1. Cytokine Modulation

Investigating the molecular mechanisms in neuroinflammation and its activation in response to Aβ and α-synuclein protein helps to identify potential therapeutic targets [197]. Recent strategies focus on targeting the pro-inflammatory cytokines for blocking and regulating their activities. Approaches such as cytokine targeting, anti-TNF antibodies, recombinant TNF and IL-1 antagonist, anti-IL-1 and IL-6 antibodies have emerged progressively [198]. However, blocking cytokines systemically faces challenges due to their pleiotropic effects in organs beyond the brain. Rather than common inhibition of cytokines, site-specific inhibition helps maintain the cytokine activities and functions in other cell types and organs [198]. Clinical studies will be essential to evaluate the efficacy and safety of cytokine therapy in human populations. Cytokine inhibitors can suppress inflammatory signaling activated by Aβ and α-synuclein. However, the outcomes are limited due to the pleotropic roles of cytokines and the risk of immunosuppression. Site-specific cytokine inhibition may overcome this challenge.

### 7.2. Overcoming the Blood–Brain Barrier

Another significant advancement in treating neurodegeneration is the development of non-invasive drug delivery across the BBB [199]. Mostly, the drugs targeting the brain tumors and psychiatric disorders face a challenge in crossing the BBB and reaching the intended site of action in the brain [200]. Despite failures of drugs at clinical stages, researchers and clinicians developed strategies to bypass or penetrate the BBB effectively [202]. In vitro BBB models have been developed based on stem cells, where induced pluripotent stem cells (iPSCs) are differentiated into induced brain microvascular endothelial cells (iBMEC) that act as a BBB model [201]. Three-dimensional extracellular matrix, transwell models, microfluidic models, 3D bioprinting, spheroids, and organoids are other static 3D environments developed to study neuroinflammation and neurodegeneration [203]. The BBB permeability remains a significant barrier for drug molecules. Advanced methods such as focused ultrasound, receptor-mediated transcytosis, and nanocarriers can deliver drugs specifically into brain regions without disrupting the BBB function.

### 7.3. The Gut–Brain Axis

Another important connectivity to the brain is the gut–brain axis. Gut dysbiosis drives systemic inflammation, promotes microglial activation, and exacerbates Aβ/tau pathology. Recent interventions using probiotics, prebiotics, and microbiome-derived metabolites demonstrated cognitive improvements in early studies. The relationship between gut permeability and neurological disorders is mediated through the gut–brain axis. Any dysbiosis in gut microbiota can cause systemic inflammation that exacerbates neuroinflammation. Manipulating gut microbiota using prebiotics and probiotics can help alter brain function. Targeting the gut–brain axis is a recent advancement in therapeutic strategies for preventing and treating neurodegenerative diseases like AD, PD, and multiple sclerosis [204,205].

### 7.4. Digital and Biomarker Technologies

The ultra-modern health care industries have developed more technologically advanced mobile-based applications and trending inventions for quick diagnosis and improvement of patient care. Digital tools like artificial intelligence (AI), machine learning algorithms, smartphone apps, and wearable devices are the most recent tools that can be able to detect subtle changes in cognitive function and behavior, and indicate the presence of dementia. Modern AI algorithms even identify AD before the onset of symptoms [209]. Smartphone apps help assess the spatial navigation skills of patients with dementia. Wearable devices help monitor physical activities and sleep patterns, which are potential indicators of dementia [12]. In treating VaD, currently risk management steps including combination with new therapies using phosphodiesterase inhibitors for cerebral perfusion and NLRPs inflammasome inhibitors for neuroinflammation, senolytics for cellular senescence, and remyelination agents for white matter repair have been introduced [39].

### 7.5. Pharmacological Interventions

Recent studies underscore the roles of non-steroidal anti-inflammatory drugs (NSAIDs) in modulating neuroinflammation associated with neurodegenerative disorders like AD and PD. NSAIDs inhibit cyclooxygenase enzymes (COX-1 and COX-2) and reduce pro-inflammatory prostaglandins, which contribute to neuronal damage [210]. An animal study with NSAID exposure showed a reduction in the formation of Aβ plaques in the brains of mice [211]. Certain prospective cohort studies have shown that dementia risk decreased with long-term use of NSAIDs. These studies discussed that prolonged use of NSAIDs, not intensive exposure, may suppress neuroinflammatory and help to reduce dementia [210,212]. Inhibitors of the NLRP3 inflammasome are another promising agent in mitigating neuroinflammation. As NLRP3 is a key immune regulator, inhibitors of NLRP3 such as MCC950, cy-09, and OLT1177 are proven to have neuroprotective effects [213] and alleviate cognitive impairment, and also support cognitive development in AD [214]. Although the clinical trials are still in an early phase, these NLRP3 inhibitors show potential effects in reversing cognitive decline by regulating microglial activation and reducing neuroinflammatory cascades. Minocycline, a tetracycline derivative, has been used as a promising immunomodulatory agent in attenuating neuroinflammation by inhibiting microglial activation and releasing pro-inflammatory cytokines. Four weeks of intraperitoneal injection of minocycline in twelve-month-old transgenic mice expressing vasculotropic Dutch/Iowa (E693Q/D694N) mutant human Aβ precursor protein showed no effect on cerebral Aβ deposition, microvascular Aβ load, and reactive astrocyte levels remained unaffected, but a significant reduction in IL-6 and improvement in spatial learning memory were found [215]. A double-blind randomized clinical trial with 544 patients with mild AD received 400 mg/d or 200 mg/d minocycline hydrochloride for 24 months. Minocycline did not delay the progress of cognitive decline and functional impairment in patients [216]. Pharmacological interventions include acetyl-cholinesterase inhibitors, including donepezil, rivastigmine, galantamine, and NMDAR antagonist memantine [12]. In high-income countries, memory clinics are initiated for the management of patients with cognitive complaints. The memory clinics offer clinical evaluation, diagnosis, staging, treatment, and rehabilitation. Non-pharmacologic and pharmacologic treatments are now available in these health care centers to delay disability and relieve psychological stress [206].

### 7.6. Monoclonal Antibodies

Monoclonal antibodies (mAbs) represent another advanced disease-modifying therapeutic strategy for neurodegenerative disorders. Aducanumab and Lecanemab were approved by the US Food and Drug Administration. Donanemab is currently under phase II and phase II clinical trials. Anti-amyloid mAbs decrease Aβ plaques and cognitive decline. PET study revealed that Aducanumab showed marked Aβ-lowering effects. The mechanism of each mAb differs in its mode of action in targeting the Aβ fibrils. Aducanumab targets a broad range of Aβ species with greater affinity; Lecanemab targets protofibrils rather than Aβ plaques. Donanemab and Remternetug mAbs target pyroglutamate Aβ in the plaques [217]. However, there are also risks of treatment with mAbs that have to be considered, like the occurrence of amyloid-related imaging abnormalities, brain volume loss, headaches, seizures, confusion, and, in extreme conditions, fatal outcomes. In such cases, the clinicians must be aware of the risk profile of patients and their genotypes [218,219]. mAbs therapies require more advanced medical infrastructures for safe management, including progressive diagnostic methods like amyloid PET, CSF Aβ measures, MRI resources, infusion centers, and intensive care units [217]. The intricate signaling networks and cellular signals provide opportunities for developing therapeutics. However, the heterogeneity of inflammatory responses across diseases and patients necessitates personalized and timed interventions.

### 7.7. Natural Compounds and Nanotechnology Approaches

In addition to pharmacological agents, natural compounds such as curcumin, resveratrol, quercetin, and epigallocatechin exhibit anti-inflammatory antioxidant properties and improve mitochondrial function by modulating NF-κB, Nrf2, and PI3K/AKT pathways [220]. Plant-derived neuroprotective chemicals, including organosulfur, flavone, alkaloid, and polyphenols, also prevent neurodegeneration. Other natural substances, such as luteolin, hesperidin, and genistein, have shown anti-neurodegenerative effects. But mostly, these natural compounds still face a major obstacle in crossing the BBB into the brain. Advanced delivery systems, including nanoemulsions, nanocarriers, nanomicelles, nanogels, and other nano-supported lipid carriers, can enhance the stability and bioavailability of these compounds in the brain [221]. Although challenges persist in transforming preclinical trials into clinical efficacy, taken together, all these therapeutic strategies possess a multifaceted approach to attenuating neuroinflammation and neurodegeneration by targeting various targets and signaling pathways. Mostly pre-clinical evidence, small human trials, and pilot studies, as well as systematic reviews, are now available regarding the treatment with NSAIDs, cytokine modulation, BBB models, and gut–brain axis modulation. 

## 8. Challenges and Future Directions

The research stage of development in treating multiple dementias through shared pathways is complex due to translational challenges. The spectrum of dementia disorders shares neurochemical mechanisms that provide both opportunities, as well as gaps in therapeutic interventions [252]. Another significant challenge is to diagnose and monitor the disorders before they progress into the dementia stage. Although various biomarkers are available to distinguish between different forms of dementia, they facilitate more accurate treatments for each type. Transforming biomarkers into clinical practice is more challenging. While advancements in biomarker research and understanding of the molecular mechanisms underlying dementia offer promising pathways, translating these findings into clinical practice remains an ongoing challenge. Future directions should focus on personalized treatment strategies, comprehensive research frameworks, and holistic management approaches to effectively bridge the existing gaps. Future research in dementia risk reduction requires a multidomain approach, population health approaches, brain health-improving methods, and person-centered outcomes that improve cognition [207]. The International Research Network on Dementia Prevention was established to bring together researchers and policymakers working on dementia prevention and dementia risk reduction. The goal of this network is to develop research into practice to reduce the risk of dementia worldwide [208]. The translational gaps are not only due to the application of pharmacological interventions, but also in the management of dementia and comorbid conditions.

Although neuroinflammation is increasingly recognized as a common feature across several NDs, important contradictions exist. Though heterogeneity in different brain regions reflects multiple interacting factors, cell types, brain region differences, neurotransmitter systems, connectivity between the cells, biochemical and metabolic differences, the core inflammatory signaling pathways and their mediators (e.g., cytokines, complement activation, NF-κB-driven cascades) are common across the CNS. Therefore, the inflammatory machinery may be fundamentally similar, but the spatial manifestation of these responses varies according to regional vulnerability, local cellular composition, and disease-specific protein pathology.

## 9. Conclusions

In summary, the neuroimaging studies and biomarker profiles across ADD, LBD, FTD, and VaD provide compelling evidence that dysregulated neuroinflammatory processes are the shared pathological hallmark of dementias. The difficulty in treating the neurodegeneration is not only linked to diagnostic challenges, but is also due to its multispecific pathological features involving multiple components at a time, like oxidative stress, mitochondrial dysfunction, endothelial dysfunction, glymphatic damage, nerve injury, demyelination, neuronal network loss, and neuronal cell death. Thus, the shared network of inflammatory events deepens our understanding of dementia pathogenesis and opens avenues for new therapeutic possibilities. Addressing the challenges in dementia requires multifaceted approaches involving early diagnosis, accurate prognosis, monitoring, and therapies promoting the BBB integrity, remyelination, targeted anti-inflammatory therapies, cytokine therapies, and combination therapies. Considering the nature of pathological features and neurodegeneration, depending on the context, discussing the challenges in therapeutic intervention helps to overcome limitations.

## Figures and Tables

**Table 1:** Important gene mutations are involved in different types of dementia and neurodegeneration.

Genes	Effect of Gene Mutation	References
Alzheimer’s disease dementia		
*APOE*	*APOE* encodes the glycoprotein apolipoprotein, which acts as a major lipid carrier in the brain and the periphery. The *APOE4* allele is a major genetic risk factor for AD and other dementias. Human *APOE4* homozygotes show an approximately 15-fold increased risk of AD, while *APOE4* carriers exhibit earlier Aβ deposition, greater overall aggregation, wider cortical distribution, and reduced dendritic spine density.	[125]
*APP*	Although APP plays an important function in neuronal development, synapse formation, and repair, mutations in APP are pathogenic. APP mutation is implicated in familial AD. Such mutations cause increased production of Aβ and promote excessive endocytosis of APP. Alternatively spliced forms of *APP* produce APP695, APP751 which undergo α- and β-secretase cleavage, thereby influencing Aβ production.	[126]
*TREM2*	Mutations and polymorphisms in *TREM2* increase the risk of AD. The normal function of TREM2 involves limiting tau-mediated damage. Pathogenic variants of TREM2, such as R47H, R62H, or H157Y, are associated with an increased risk of AD. The R47H variant can reduce the proliferation of microglia around senile plaques and promote the spread of Tau. Reduced TREM2 activity can reduce microglial response to tau. In different stages of AD, TREM2 appears to have stage-dependent effects on Tau pathology.	[127]
*PSEN 1*/*PSEN 2*	Autosomal dominant mutations in *PSEN1* and *PSEN2* cause familial AD. Mutations of *PSEN 1* and *PSEN2* cause early-onset Alzheimer’s disease by disrupting gamma-secretase activity, thereby altering APP cleavage to favor the production of toxic Aβ_42_ peptide. Mutant presenilins can increase overall Aβ production.	[128]
*CLU*	Mutations in *CLU* are a genetic risk factor of AD. *CLU* is involved in Aβ processing, deposition, clearance, tau protein pathology, neuroinflammation, and lipid metabolism. CLU is an apolipoprotein that regulates the complement pathway, participates in microglial activation, and binds to the TREM2 receptor in microglia. As a glycoprotein, CLU, along with chaperones bind to Aβ and helps in clearing Aβ fibrils and peptides. Mutations in *CLU* disturb Aβ clearance and exacerbate pathology.	[129]
*CR1*	CR1 is a membrane receptor for C3b, which controls complement activation. In AD, complement activation is associated with inflammation and glial cell activation. C3b is a complement factor that colocalizes with amyloid plaques and tangles. Mutations in *CR1* impair its ability to bind C3b and propagate the complement pathway.	[130]
*PICALM*	PICALM is a phosphatidylinositol-binding clathrin-adaptor protein that plays a critical role in clathrin-mediated endocytosis, autophagy, and Tau pathology. The *PICALM* gene is a genetic susceptibility locus for late-onset Alzheimer’s disease. Genetic changes in *PICALM* disrupt the synapse vesicle cycling and increase the risk of AD. PICALM can influence APP processing through endocytotic pathways and change levels of Aβ.	[131,132]
*SORL1*	The neuronal sortilin-related receptor SORL1 is known to be involved in the trafficking and processing of APP. Rare loss-of-function *SORL1* alleles have been linked to late-onset Alzheimer’s disease (AD), primarily through their effects on Aβ homeostasis and impaired APP trafficking.	[133]
Vascular dementia		
*NOTCH3*	NOTCH receptors are predominantly present in vascular smooth muscle cells of small arteries. The *NOTCH3* gene mutation causes CADASIL. CADASIL is characterized by VaD or VCID. A mutation in the *NOTCH3* gene affects the extracellular domain of the receptor, leading to protein misfolding and receptor aggregation.	
*APOE*	*APOE* carriers exhibit greater cognitive impairment compared to other *APOE* alleles. *APOE4* allele causes cholesterol dysfunction, inflammation, metabolic dyshomeostasis, BBB breakdown, and induces cerebrovascular damage and increase risk of VCID. Both *APOE2* and *APOE4* alleles contribute to amyloid accumulation in the parenchymal and meningeal cerebrovascular system.	[125,134]
*COL4A1*	*COL4A1* encodes the type IV collagen alpha protein associated with ischemic and hemorrhagic stroke. Mutations in the *COL4A1* gene cause cerebral small vessel arteriopathy and cerebral hemorrhage.	[135]
*VEGF*	The *VEGF* gene encodes a heparin-binding protein, VEGF are important signaling protein involved in the blood vessel growth, permeability, and maintenance of both vascular and neural cells. Reduced VEGF levels have harmful consequences for both vascular health and cognitive wellbeing. VaD and MCI patients showed changes in VEGF levels in blood and CSF. VEGF may serve as a predictive marker for identifying VaD	[136]
*SREBF-2*	SREBP-2 plays a key role in cholesterol synthesis and is associated with the regulation of certain genes that contribute to amyloid and tau proteins. Lowering SREBP levels reduces amyloid production, hypercholesterolemia, and associated cognitive impairment.	[137]
Lewy body dementia		
*SNCA*	Mutations in *SCNA* are the specific cause of LBD. *SNCA* mutations exacerbate α-Syn aggregation and disease severity. Aggregates disrupt neuronal functions. A mutation in *SCNA A53T*, *E46K* can alter the properties of the α-synuclein protein, promote its aggregation, and fibril formation.	[138]
*PSEN 1/PSEN 2*	The *PSEN 2 A85V* mutation is associated with LBD. Other mutations of *PSEN2*—R71W and R62H—were identified in LBD patients. *PSEN1* mutation is associated with increased accumulation of α-synuclein. LB pathology in the amygdala showed a higher frequency of *PSEN1* mutations than of *PSEN2* mutations.	[139,140]
*APOE*	*APOE4* allele carriers are more likely to increase in LB pathology. *APOE4* regulates α-synuclein pathology and exacerbates its toxic effects. APOE4 regulates α-synuclein pathology, independently of amyloid deposition.	[141]
*GBA*	*GBA1* encodes the lysosomal enzyme glucocerebrosidase responsible for the breakdown of glucocerebroside into glucose and ceramide. *GBA1* mutations reduce the enzyme glucocerebrosidase activity, resulting in glucocerebroside accumulation inside the lysosome. This accumulation alters lysosomal pH, impairs hydrolase activities, and affects α-synuclein metabolism.	[141]
Frontotemporal dementia		
*GRN*	The *GRN* gene encodes the progranulin protein, which is the common cause of FTD. Progranulin helps regulate lysosomal homeostasis, inflammatory processes, neural function, and differentiation under normal and pathological conditions. Mutations in *GRN* reduce progranulin levels and hinder its cellular functions. In FTD cases, 5 to 10% are caused by mutations in the *GRN* gene.	[141,142]
*C9ORF72*	Approximately 25% of familial FTD cases are attributed to hexanucleotide repeat expansion mutations in the chromosome 9 open reading frame 72 (*C9ORF72*) gene. *C9ORF72* pathology is often associated with TDP-43 pathology. Loss of function of C9ORF72 induces neurodegeneration. FTD cases frequently exhibit expanded repeats in *C9ORF72*, with more than 30 repeats considered pathogenic.	[143]
*MAPT*	Mutations in *MAPT* lead to FTD by altering tau splicing, impairing microtubule binding, promoting hyperphosphorylation, and driving misfolding and aggregation of tau.	[144]
*CHMP2B*	CHMP2B is essential for membrane deformation and endosomal maturation. Mutations in *CHMP2B* reduce the number of endolysosomes and significantly impair their trafficking within neuronal dendrites, leading to lysosomal storage pathology and ultimately contributing to the development of FTD.	[145]
*SQSTM1*	The *SQSTM1* gene, located on chromosome 5, encodes p62, a protein involved in autophagy, protein degradation, and NF-κB activation. Mutations in *SQSTM1* alter the SQSTM1 protein (p62) aggregation resultant pathogenesis of FTD.	[146]

*APOE*—apolipoprotein E; Aβ—amyloid beta; *APP*—amyloid precursor protein; AD—Alzheimer’s disease; *TREM2*—triggering receptor expressed on myeloid cells 2; *PSEN1*—presenilin 1; *PSEN2*—presenilin 2; *CLU*—clusterin; *CR1*—complement receptor 1; *PICALM*—phosphatidylinositol binding clathrin in assembly protein; *SORL1*—sortilin-related receptor; *NOTCH3*—neurogenic locus notch homolog protein 3; CADASIL—cerebral autosomal dominant arteriopathy with subcortical infarcts and leukoencephalopathy; VaD—vascular dementia; VCID—vascular cognitive impairment and dementia; *COL4A1*—collagen type IV alpha 1 chain; *VEGF*—vascular endothelial growth factor; MCI—mild cognitive impairment; CSF—cerebrospinal fluid; *SREBF-2*—sterol regulatory element binding protein 2; LB—Lewy bodies; LBD—Lewy body dementia; SCNA—alpha synuclein; *GBA*—glucocerebrosidase; *GRN*—progranulin; FTD—frontotemporal dementia; *C9ORF72*—chromosome 9 open reading frame 72; TDP-43—TAR DNA binding protein; *MAPT*—microtubule-associated protein tau; *CHMP2B*—charged multivesicular body protein 2B; *SQSTM1*—sequestosome 1; *SNCA*—alpha-synuclein; NF-κB—nuclear factor κappa B.

**Table 2:** Comparison of neuroinflammation profiles across different types of dementias.

Features	Alzheimer’s Dementia	Lewy Body Dementia	Frontotemporal Dementia	Vascular Dementia
Initiating factor	Aβ aggregation, Tau pathology, Gene mutations	α-aggregates as LB, gene mutations	TDP-43 aggregates, Tau pathology	Cerebral hypoperfusion, ischemia, microinfarct, blood vessel damage, stroke
Neuroinflammatory Pathways	Activation of NF-κB, NLRP3 inflammasome, TLR2, TLR4, NLR, MAPK, JAK/STAT, MAPK, cGAS–STING pathways	Activation of TLR, NF-κB, NLRP3 inflammasome, JAK/STAT, MAPK, MyD88, and complement system-mediated pathways	Activation of NF-κB, NLRP3 inflammasome, and TLR4 pathways	Activation of NF-κB, NLRP3 inflammasome, JAK/STAT, HIF-1α Pathway
Microglial activation	Highly activated by Aβ plaques and tau protein aggregates, oxidative Stress.	Activated by oxidative Stress, α-synuclein protein aggregates	Activated by Gene mutations, TDP-43, and Tau protein aggregates	Activated by hypoxic, ischemic injury, oxidative Stress, white matter injury, myelin damage, and Amyloid co-pathology
Astrocytes	Reactive astrocytosis, cytokine release	Astrocytosis reacts but less than AD	High astrocytosis in tau-mediated FTD	Reactive astrocytes are found near infarct sites and scars
Peripheral immune response	Compromised BBB in the later stage	Less BBB damage than AD and VaD	BBB damage	Massive BBB breakdown
Pro-inflammatory mediators	Increased TNF-α, IL-1β, IL-6, IL-8, IL-18, chemokines CCL2, CCL3, CXCL8, NF-κB transcription factors	Increased TNF-α, IL-1β, IL-6; IL-10, MHCII	Upregulated TNF-α, IL-6, IFN-γ, complement activation	Elevated IL-1β, TNF-α, MMP2, ROS, RNS, HIF-1α
Spatial distribution	Hippocampus, entorhinal cortex	Midbrain, Brainstem, limbic system, cingulate cortex, cholinergic basal brain	Frontal and temporal lobes	Subcortical white matter, basal ganglia, watershed zones.
Neuronal loss	Glutamatergic Cholinergic, hippocampal neurons	Dopaminergic, cholinergic, noradrenergic neurons	GABAergic neurons, Frontal/temporal pyramidal neurons	White matter axons, oligodendrocytes, and cortical neurons

Aβ—amyloid beta; α-syn—alpha-synuclein; NF-κB—nuclear factor kappa B; NLR—NOD-like receptor family; NLRP3—NOD-like receptor family, pyrin domain containing 3; TLR2, TLR4—toll-like receptor 2, 4; MAPK—mitogen-activated protein kinase; JAK/STAT—Janus kinase/signal transducer and activator of transcription; MyD88—myeloid differentiation primary response protein 88; cGAS–STING—cyclic GMP-AMP synthase-stimulator of interferon genes; HIF-1α—hypoxia-inducible factor; TDP-43—TAR-DNA binding protein 4; TNF-α—tumor necrosis factor-alpha; IL—interleukin; IL-1β—interleukin 1 beta; ROS—reactive oxygen species.

**Table 3:** Categories of biomarkers according to the method of analysis.

Ex Vivo (Fluid Markers)		In Vivo	Post-Mortem (Tissue-Based Examination)
Blood	CSF	Neuroimaging	
Plasma p-tau (181,217,231) Plasma Aβ42/40 ratio Plasma NfL Plasma GFAP Inflammatory cytokines Inflammatory Chemokines Serum progranulin	CSF Aβ42/40 CSF p-tau (181,217) CSF total tau CSF NfL CSF TREM2 CSF albumin ratio CSF GFAP, YKL-40	PET (Aβ-PET, Tau-PET, TSPO-PET) MRI DTI fMRI SPECT	Histopathology of Aβ plaques Staging of NFTs Lewy body pathology Microglial morphology changes Mitochondrial abnormalities Lysosomal pathology

NfL—neurofilament light chain; GFAP—glial fibrillary acidic protein; CSF—cerebrospinal fluid; YKL-40—tyrosine, lysine, leucine-40; PET—positron emission tomography; MRI—magnetic resonance imaging; DTI—diffusion tensor imaging; fMRI—functional MRI; SPECT—single-photon emission computed tomography; Aβ—amyloid beta.

## Data Availability

No new data were created or analyzed in this study.

## Abbreviations

AD	Alzheimer’s disease
ALS	Amyotrophic lateral sclerosis
APOE	Apolipoprotein E
APP	Amyloid precursor protein
Aβ	Amyloid β
BBB	Blood–brain barrier
BDNF	Brain-derived neurotrophic factor
bvFTD	Behavioral variant frontotemporal dementia
CADASIL	Cerebral autosomal dominant arteriopathy with subcortical infarcts and leukoencephalopathy
CCL	Chemokine C-C motif ligand
CCR	C-C chemokine receptor
CNS	Central nervous system
COX1,2	Cyclooxygenase 1,2
CREB	Cyclic response element binding protein
CSF	Cerebrospinal fluid
CXCL	chemokine (C-X-C motif) ligand
DAMP	Damage-associated molecular patterns
EOAD	Early-onset Alzheimer’s disease
FTD	Frontotemporal dementia
GFAP	Glial fibrillary acidic protein
GRN	Progranulin
GSK3 β	Glycogen synthase kinase-3β
GWAS	Genome-wide association studies
iBMEC	Induced brain microvascular endothelial cells
IDE	Insulin-degrading enzyme
IL	Interleukin
JaK	Janus kinase
LB	Lewy body
LBD	Lewy body dementia
LOAD	Late-onset Alzheimer’s disease
LPS	Lipopolysaccharide
LRRK2	Leucine-rich repeat kinase 2
MAP	Microtubule-associated protein
MAPK	Mitogen associated protein kinase
MAPT	Microtubule-associated protein tau
MCI	Mild cognitive impairment
MHC	Major histocompatibility complex
MMP	Matrix metalloproteinase
mAb	Monoclonal antibodies
MyD88	Myeloid differentiation factor 88
NFT	Neurofibrillary tangles
nfvPPA	Non-fluent variant primary progressive aphasia
NF-κB	Nuclear factor κappa B
NLRs	NOD-like receptors
NLRP3	NOD-like receptor family, pyrin domain containing 3
NSAIDs	Non-steroidal Anti-Inflammatory Drugs
PAMP	Pathogen-associated molecular patterns
PD	Parkinson’s disease
PDD	Parkinson’s disease dementia
PET	Positron emission tomography
PNS	Peripheral nervous system
PSEN	presenilin
SCNA	Synuclein A
STAT	Signal transducer and activator of transcription
svPPA	Semantic variant primary progressive aphasia
TDP43	TAR-DNA binding protein 43
TGF-β	Tumor growth factor β
TLRs	Toll-like receptors
TNF-α	Tumor necrosis factor α
TREM2	Triggering Receptor Expressed on Myeloid Cells 2
TSPO	Translocator protein
VaD	Vascular dementia
VCI	Vascular cognitive impairment
VCID	Vascular cognitive impairment and dementia
VEGF	Vascular endothelial growth factor
WHO	World Health Organization
